# Cross-reactivity of pollen and food allergens: soybean Gly m 4 is a member of the Bet v 1 superfamily and closely resembles yellow lupine proteins

**DOI:** 10.1186/2045-7022-1-S1-P6

**Published:** 2011-08-12

**Authors:** Paul Rösch, Hanna Berkner, Philipp Neudecker, Diana Mittag, Barbara Ballmer-Weber, Peter Lehn, Kristian Schweimer, Stefan Vieths

**Affiliations:** 1 Universität Bayreuth, Biopolymere, Bayreuth, Germany; 2 University Hospital, Dermatology, Zürich, Switzerland; 3 Universitätsklinikum Regensburg, Klinische Chemie und Laboratoriumsmedizin, Regensburg, Germany; 4 Paul-Ehrlich-Institut, Allergologie, Langen, Germany

## 

In many cases, patients allergic to birch pollen also show allergic reactions after ingestion of plant-derived food. This observation is explained on the molecular level by cross-reactivity of IgE antibodies induced by sensitization to the major birch pollen allergen Bet v 1 with homologous food allergens. As IgE antibodies recognize conformational epitopes, a precise structural characterization of the allergens involved is necessary to understand cross-reactivity and thus to develop new methods of allergen-specific immunotherapy for allergic patients. Here we present the three-dimensional solution structure of the soybean allergen Gly m 4, a member of the superfamily of Bet v 1 homologous proteins and a cross-reactant with IgE antibodies originally raised against Bet v 1, as shown by immunoblot inhibition and histamine release assays. Although the overall fold of Gly m 4 is very similar to that of Bet v 1, the three-dimensional structures differ in detail. The Gly m 4 local structures that display those differences are also found in proteins from yellow lupine. The three-dimensional structure of Gly m 4 in combination with immunological data allows us to propose surface patches that might represent cross-reactive epitopes.

